# Sugar Detection in Aqueous Solution Using an SMS Fiber Device

**DOI:** 10.3390/s23146289

**Published:** 2023-07-11

**Authors:** Nailea Mar-Abundis, Yadira Aracely Fuentes-Rubio, René Fernando Domínguez-Cruz, José Rafael Guzmán-Sepúlveda

**Affiliations:** 1 Centro de Innovación Tecnológica en Eléctrica y Electrónica, Universidad Autónoma de Tamaulipas, Carr. a San Fernando Cruce con Canal Rodhe S/N., Reynosa 88779, Tamaulipas, Mexico; a2183720052@alumnos.uat.edu.mx (N.M.-A.); rfdominguez@docentes.uat.edu.mx (R.F.D.-C.); 2 Center for Research and Advanced Studies of the National Polytechnic Institute (CINVESTAV Unidad Monterrey), Vía del Conocimiento 201, Parque de Investigación e Innovación Tecnológica km 9.5 de la Autopista Nueva al Aeropuerto, Apodaca 66600, Nuevo León, Mexico; jose.guzmans@cinvestav.mx

**Keywords:** fiber optics sensor, multimode interference, sugar concentration, sucrose, fructose, glucose

## Abstract

We report on the fabrication and testing of a fiber optics sensor based on multimodal interference effects, which aims at the detection of different types of sweeteners dissolved in water. The device, which has a simple structure, commonly known as the SMS configuration, is built by splicing a segment of commercial-grade, coreless multimode fiber (NC-MMF) between two standard single-mode fibers (SMFs). In this configuration, the evanescent field traveling outside the core of the NC-MMF allows the sensing of the refractive index of the surrounding media, making it possible to detect different levels of sugar concentration. The optical sensor was tested with aqueous solutions of glucose, fructose, and sucrose in the concentration range from 0 wt% to 20 wt% at room temperature. The proposed device exhibits a linear response with a sensitivity of 0.1835 nm/wt% for sucrose, 0.1687 nm/wt% for fructose, and 0.1694 nm/wt% for glucose, respectively, with a sensing resolution of around 0.5 wt%. Finally, we show that, despite having similar concentration behavior, some degree of discrimination between the different sugars can be achieved by assessing their thermo-optical response.

## 1. Introduction

Having a healthy, well-balanced diet helps to reduce the risks of obesity and developing medical conditions such as diabetes, heart illness, strokes, osteoporosis, and some types of cancer [1,2,3,4]. Sugar consumption plays a significant role in a balanced and nutritional diet, as it should be neither omitted nor exceeded in the daily diet. Sugar, along with other nutrients (proteins, fats, and vitamins), provide energy to carry out the performance of different organs whose functionality is essential for physiological functions [5,6]. The World Health Organization (WHO) suggests that sugars should make up to 5% of the total energy intake per day because, when combined with additional factors such as low physical activity, genetic predisposition, or excessive consumption, it can lead to hypoglycemia, diabetes, and cardiovascular diseases, among others [7,8].

Due to its importance, many clinical devices exist that are available in the market for measuring the levels of sugar in the blood. Nevertheless, the measurement of sugar content is also critical in the food industry, being one of the most important variables for quality control [9,10,11]. In this nonclinical context, optical detection strategies have proven suitable for the detection and quantification of the sugar content due to their potential for noninvasive, fast, and sensitive measurements [12,13].

Several approaches have been reported to measure sugar concentration, such as Raman spectroscopy coupled with neural networks [14]; the monitoring of the refractive index (RI) via reflectometric measurements [15]; induced fluorescence measurements [16,17]; and techniques based on radiofrequency [18], among others. 

Aiming at real-time operation, optical fiber-based strategies have been proposed for glucose detection due to well-known properties such as excellent biocompatibility, chemical inertness, compact size, immunity to electromagnetic interference, and the potential for remote sensing and real-time monitoring. In the literature, there are currently exhaustive reviews that summarize the main contributions focused on the measurement of sugars. For example, in Ref. [19], an interesting overview is presented on different sensors for measuring glucose based on optical fibers, some including invasive, minimally invasive, and noninvasive techniques. Also, this report compares some important parameters such as the kind of fiber used, the surface or immobilizing structure, detection and dynamic ranges, response time, and sensitivity. In Ref. [20], the authors describe some chemical principles of detection, including the oxidation reaction and covalent binding. These phenomena are related to sugar concentrations and the RI, which are used in methods based on surface plasmon resonance (SPR), fiber grating, and fluorescence techniques. Wang et al. [21] collected the highlighted reports on fiber optic chemical sensors and biosensors published between 2015 and 2019. This overview covers sensors for gases and vapors; humidity and water fraction; pH values, ions, and salinity; and sensors applied for organic species. For the last one, the authors examined the main configurations reported in recent years for glucose and other saccharides.

In terms of non-conventional cylindrical waveguides, plastic optical fibers have been reported for sugar measurements in combination with SPR effects [22,23]. In addition, other special fiber structures have been reported, such as functionalized long-period gratings (LPGs) [24] and tilted Bragg gratings [25]. These methods can provide detailed information; however, they often require extensive complex procedures, special fiber preparation, or a significant amount of peripherical instrumentation. Furthermore, the use of special fibers, such as photonics crystal fibers (PCFs) filled with nanowires [26] and multicore fibers [27], has been reported for glucose concentration sensing. These novel techniques can also constitute efficient sensors; nevertheless, the use of special fibers makes these sensors more expensive and harder to handle.

A particular type of fiber optics sensor that has been demonstrated to be useful in several fields of science and engineering is that based on multimode interference (MMI), which occurs when a segment of a multimode fiber (MMF) is spliced between two single-mode fibers (SMFs); this architecture is frequently referred to as singlemode–multimode–singlemode, or simply as SMS. This optical configuration has been reviewed extensively in several recent review articles [28,29,30,31], highlighting its advantages of being an all-fiber solution that is easy to manufacture, package, and interconnect to other fiber components. Interestingly, MMI effects have been used previously for sugar detection in integrated devices [32,33]. Additionally, a theoretical report exists where MMI effects in optical fibers were studied for glucose sensing using a structure consisting of a segment of PCF as the multimodal section [34].

In this paper, we present the fabrication and testing of an SMS sensor suitable for the measurement of the sugar content in aqueous solutions. The sensor presented has the potential to be used in the food industry for the detection of sugar level concentrations and quality control. The device proposed was built by assembling a segment of coreless multimode fiber (also known as no-core MMF, NC-MMF) between two standard SMFs. We performed the measurements using three different types of sweeteners: glucose, fructose, and sucrose. From the interaction of the evanescent field in the multimode fiber section with the surrounding media, it is possible to measure the concentration of sugar indirectly through variations of the refractive index. Moreover, we demonstrate that some degree of discrimination among sugars can be achieved by making use of the thermo-optic effects.

## 2. Theoretical Description 

### 2.1. MMI as Sensor Device

**Figure 1:**
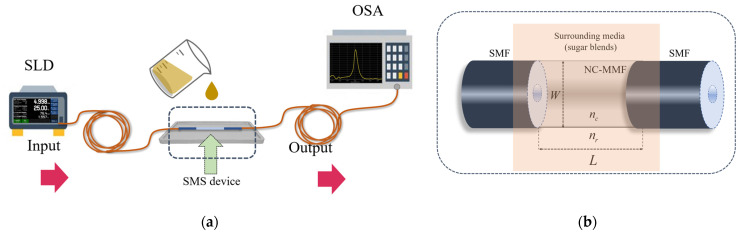
(**a**) Experimental array to test SMS device for the measurement of sugar concentrations. (**b**) Typical structure used to perform the multimodal interference effect (MMI) in optical fibers.

The well-common SMS structure of fiber optic MMI devices consists of an MMF spliced between two SMFs [28,29,30,31]. Typically, these sensors are tested in transmission using the experimental setup shown in Figure 1a, where a broad spectrum is fed into the sensor, and the output spectrum is measured with an optical spectrum analyzer; the setup will be discussed in more detail below. Moreover, if the cladding of the MMF is removed or absent, as in the case of an NC-MMF, then the structure can be used as the sensor of their surrounding media [31], as it is shown in Figure 1b.

When light is coupled from an SMF to an MMF (Figure 1b), several optical modes are excited in the multimode section and will interfere with one another as they propagate. The interference of these modes along the NC-MMF can replicate the input field periodically, forming self-images of the input field. Therefore, the NC-MMF must have a specific length to reconstruct the input field at the end of the NC-MMF at a certain wavelength. If another SMF is placed after the MMF, and a broad spectrum is fed into the structure, only a specific wavelength satisfies the condition of constructive interference at the output. In this configuration, the peak wavelength, $\lambda_{peak}$, that replicates the p-th image of the input field in an MMI device is expressed as [35,36]:

(1) $$\lambda_{peak}=p\frac{n_{eff}W_{eff}^{2}}{L_{eff}}$$

where p=1, 2, 3… is the order of the input field’s self-image, and $n_{eff}$, $W_{eff}$, and $L_{eff}$ are the effective RI of the lower order modes, the effective diameter of the MMF, and the effective length of the MMF, respectively. We refer the reader to Refs. [35,36] for the full derivation of Equation (1) in the context of integrated MMI devices, and to Ref. [28] for a simplified extension to fiber optic MMI devices. The re-imaging index in Equation (1) indicates the periodic nature of the self-image formation; however, in conditions of restricted symmetry where the optical fibers are collinear, the process of MMI has a four-fold periodicity [35]. Images formed in another location are known as false images, and although they look like the input field, they have higher losses. The effective diameter accounts for the penetration of the evanescent field into the cladding, which is of the order of the wavelength; for MMFs having geometrical diameters much larger than the wavelength, as it is our case, $W_{eff}$ approximates the diameter of the core of the MMF. The effective RI implicitly depends on the RI of the cladding of the MMF, which, in our case, is the liquid sample in which the SMS structure is immersed. In this way, by following the peak of the transmitted spectrum, one can perform a measurement of sugar concentration via the variations produced in $n_{eff}$ due to the sugar content.

### 2.2. Sugars and Refraction Index Description

Sucrose, commonly known as sugar, is a disaccharide formed by the combination of glucose and fructose, and it is one of the most widely used sweeteners worldwide [37,38]. On the other hand, glucose and fructose are monosaccharides, which are the building blocks of carbohydrates. They are simple forms of sugar found in everyday foods, including fruits, vegetables, honey, and some cereals, and are commonly used as sweeteners in beverages and processed foods [39]. 

**Figure 2:**
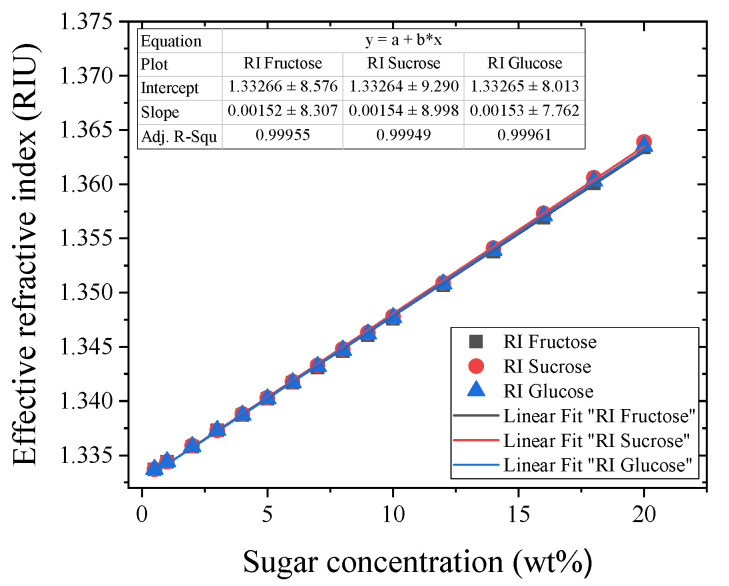
Effective refractive index of aqueous solutions of sugars as a function of the sugar concentration. The plot was made by using the numerical data reported in Ref. [42].

A common parameter used for the identification and quantification of the sugar content is the RI [39], as this optical property increases with its concentration and constitutes an important indicator of the quality of foods, beverages, fruits, and preserves [39,40]. Fortunately, the RI of aqueous solutions of different sugars has been well studied, constituting a standard reference of the US National Institute of Standards and Technology [41,42]. In particular, Ref. [42] reported the measurement of the effective RI of aqueous solutions of sucrose, fructose, and glucose at a temperature of 20 °C and a wavelength of 589 nm. These data are plotted in Figure 2, in the range of interest from 0 wt% to 20 wt%, where it can be seen that the effective RI of the aqueous solution increases linearly with the concentration ($R^{2}>0.99$ in all cases) as $n_{eff}=n_{water}+\delta n\left(C\right)$, with $n_{water}=1.3330$ and $\delta n\left(C\right)=S\times C$, where S is the slope of the linear dependence (slightly different for each type of sugar), and C is the sugar concentration (in units of wt%). Here, we note that the three different sugars have a similar concentration behavior.

In our case, the length of the NC-MMF is constant, and the optical diameter is approximately equal to the geometric one because the extent of the evanescent field (on the order of one wavelength) is small compared to the fiber diameter. In these conditions, one can see from Equation (1) that $\lambda_{peak}$ depends only on $n_{eff}$, being proportional to it, i.e., $\lambda_{peak}\propto n_{eff}$. Therefore, since $n_{eff}$ of an aqueous solution of sugar varies linearly with the sugar concentration (see Figure 2), $\lambda_{peak}$ will also increase linearly with the sugar concentration, as it will be shown later.

## 3. Methods

### 3.1. Sample Preparations

To test the sensor device, we prepared aqueous solutions of sugar using deionized water (^®^Sigma Aldrich, San Louis, MO, USA, 99% pure) and commercial brands of sucrose, fructose, and glucose in powders. In all cases, we added the sugar powder to 10 mL of deionized water; the amounts of sugar added were from 0.05 g to 2.0 g in steps of 0.15 g. In terms of weight fractions [43], the concentration range went from 0.5 wt% to 20 wt% in increments of 1.5 wt%. In terms of the molar concentration, for glucose and fructose, which have a similar molecular weight of 180.16 g/mol, the concentration range went from 0.0278 mol/L to 1.1101 mol/L; on the other hand, for sucrose (342.30 g/mol), the concentration range went from 0.0146 mol/L to 0.5843 mol/L.

### 3.2. Sensor Device

The SMS element was fabricated using SMF (SMF-28, ^®^Thorlabs, Newton, NJ, USA), with a core/cladding diameter of 8µm/125µm, respectively, and an NC-MMF (FG125LA, ^®^Thorlabs), which is a uniform silica rod with a diameter of $D_{MMF}=125\;µm$ and refractive index of $n_{r,MMF}=1.4445$. The sensor was fabricated by following a simple procedure that consisted of splicing the SMF to the NC-MMF; then, the desired length of the NC-MMF was measured with a vernier caliper, taking the splice as the zero-length; then, the NC-MMF was marked at the desired length and cleaved at the mark; finally, the second SMF was spliced. All segments were spliced using an arc fusion splicer (^®^Fujikura, San Jose, CA, USA, model FSM-60S). Based on Equation (1), the length of the NC-MMF was estimated to be L=57.3 mm for the peak wavelength to be centered at $\lambda_{peak}=1575\;nm$ for the baseline condition where the MMI device was in the air. The fabrication procedure is straightforward and robust: by doing a simple variational analysis of Equation (1), one can see that the variation in the peak wavelength produced by an error in the length of the MMF is $\delta \lambda_{peak}=\left(\frac{\partial \lambda_{peak}}{\partial L}\right)\delta L=-\left(p\frac{n_{eff}W_{eff}^{2}}{L^{2}}\right)\delta L$. Thus, by taking p=4, $n_{eff}\approx 1.444$, $W_{eff}\approx 125\;\mu m$, L=57.3 mm, and considering the standard resolution of a general purpose vernier caliper as δL=0.05 mm, the error in the peak wavelength is of only $\delta \lambda_{peak}\approx 1.4\;nm$. This simple procedure can be used to engineer MMI sensing architectures where multiple sensors operate simultaneously, each of them in their own spectral window, but all of them within the range of a single broadband light source [44]. Importantly, the NC-MMF did not require any additional coating or treatment. 

### 3.3. Experimental Setup

The optical system to test the SMS sensor is depicted in Figure 1a. In this scheme, a superluminescent laser diode (SLD, Model SLD1550S-A1, @Thorlabs) provided a broadband spectrum from 1420 nm to 1650 nm, which was launched into a commercial FC/PC patch cable. The signal was propagated into the SMS device, collected by a second patch cable, and measured with an optical spectrum analyzer (OSA, ^®^Anritsu, Morgan Hill, CA, USA, model MS9740A). The active region of the sensor was attached to a container to ensure that it was fully immersed in the aqueous solution. 

## 4. Results and Discussion

We first measured the spectral response of the MMI with the different sucrose blends, beginning with the lowest concentration. The spectral response was registered with the OSA. After each measurement, the sensor was cleaned using deionized water and dried in order to come back to the initial condition, where the spectrum peaks at 1575 nm. A similar process was used for the aqueous solutions of fructose and glucose.

**Figure 3:**
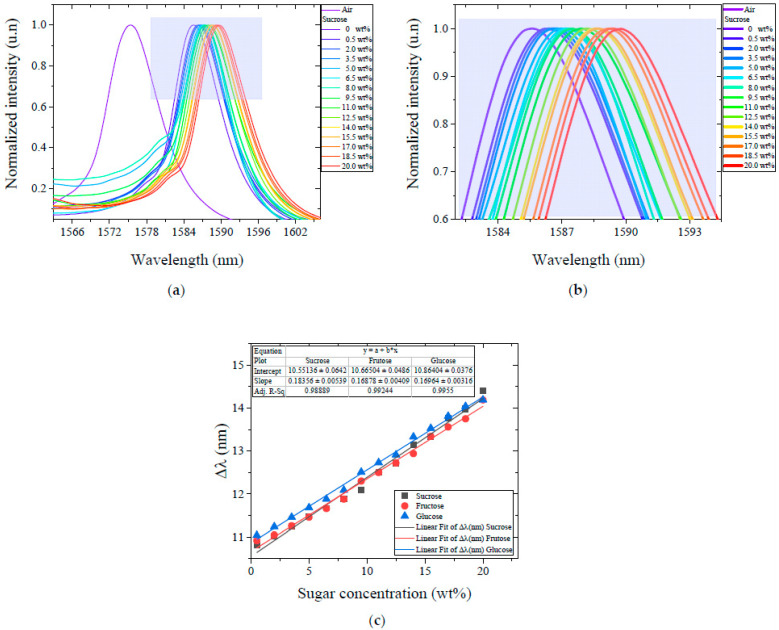
(**a**) Spectral response of the MMI sensor for water–sucrose blends. (**b**) The shaded box shows in more detail the region of the spectral change in panel (**a**), which corresponds to the NC-MMF section surrounded by each sugar type concentration. (**c**) The spectral shift of the peak wavelength, with respect to the baseline condition, as a function of the concentration of sucrose, fructose, and glucose present in the aqueous solution.

Figure 3a shows the spectral response when the sensor was immersed in different aqueous solutions with different concentrations of sucrose. The spectral shift was positive and was proportional to the concentration level from 10.81 nm to 14.41 nm with respect to the baseline condition when the sensor was in the air. A similar situation occurred when the sensor was immersed in the different concentrations of fructose and glucose; the spectral shift was positive from 10.92 nm to 14.19 nm and from 11.04 nm to 14.19 nm, respectively. Figure 3b shows in more detail the spectral shift, where it can be appreciated that the sensor responded to a concentration as low as 0.5 wt%.

To appreciate the response of the sensor for each type of sugar, the spectral shift, that is, the change in $\lambda_{peak}$, was plotted as a function of concentration, as shown in Figure 3c. In all cases, the spectral shift was measured with respect to an initial condition where the SMS structure was in the air. The results show that $\lambda_{peak}$ shifted linearly towards longer wavelengths proportionally to the concentration present in the solution, as expected from the fact that the effective RI of sugar-aqueous solutions increases linearly with the concentration [42] (see Figure 2). Experimental results indicate a sensitivity of 0.1835 nm/wt% for sucrose, 0.1687 nm/wt% for fructose, and 0.1694 nm/wt% for glucose. Equivalently, in terms of the molar concentration, the sensitivity is 3.3222 nm/(mol/L), 3.3278 nm/(mol/L), and 6.6455 nm/(mol/L) for fructose, glucose, and sucrose, respectively. The resolution of the OSA used in the experiments is 0.2 nm, which results in a resolution for the measurements (the limits of detection) of 0.0602 mol/L, 0.0601 mol/L, and 0.0301 mol/L for fructose, glucose, and sucrose, respectively. If seen as a refractometer, our sensor has a similar sensitivity to other MMI fiber optic sensors where a bare NC-MMF is used [31]. From the standpoint of a sugar sensor, our sensor has a similar sensitivity to other mode interference sensors but is much lower than that exhibited by more sophisticated sensors based on surface plasmon resonance or tilted fiber gratings with functionalized metallic coatings [20]. In this regard, it should be noted that, in our approach, a short section of bare, untreated NC-MMF was used that did not require any processing; ultimately, the sensitivity of the sensor presented can be improved by implementing the enhancing mechanisms such as by tapering, etching, or coating [29,30].

Regarding the spectral width, we note that the spectrum slightly broadens as the sugar concentration is increased, but the changes are subtle; the bandwidth increases by about 0.5 nm across the entire range of the experiments (from 9 nm in water to 9.5 nm at the maximum concentration of sugar, approximately).

The effective RI of the lower order modes can be estimated from the spectral shifts by doing a simple variational analysis where the length of the NC-MMF is assumed to be invariant, and the change in the optical diameter is neglected, i.e., $n_{eff}^{\prime }\approx \left(\lambda_{peak}+\delta \lambda_{peak}\right)L_{NCF}/\left(p\;W_{NCF}^{2}\right)$. Moreover, expressions of the effective RI can be found in the literature where the RI of the cladding appears explicitly [45,46]. By solving those expressions numerically, one could attempt to retrieve the actual RI of the cladding (of the aqueous solution in our case and, from there, the concentration of sugar).

To describe the error level and the measurement’s repeatability, three representative samples of sucrose, fructose, and glucose were selected arbitrarily, with concentrations of 6.5%, 12.5%, and 18.5%. For each selected sample, the spectral shift was registered following the procedure described above, which includes cleaning with deionized water and drying. This process was performed in triplicate for each sample of each type of sugar. The standard deviations that were obtained for all these repetitions are illustrated in Table 1. From these values, we observe that the measurements for concentrations of 6.5% and 12.5% are consistent in each repetition. For the case of the 18.5% concentration, we note a variation, probably attributed to the fact that the sugar level used was close to the limit of the solution. 

**Figure 4:**
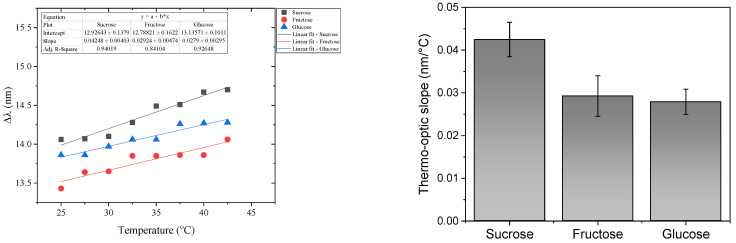
The thermal effect reported for each sugar type using a concentration of 18.5%. Measurements were performed in the temperature range from 25 °C to 42.5 °C in increments of 2.5 °C.

The thermo-optic effects were also investigated, and for this propose, we carried out the following test. From the initial conditions in the air, the sensor was immersed in a sample using a concentration of 18.5% sucrose. The container was placed on a hot plate (^®^IKA Wilmington, NC, USA, Model HS7), and the temperature was increased from 25 °C to 42.5 °C in increments of 2.5 °C. In this process, the sensor was kept inside the temperature chamber. At each temperature increase, the sample was stabilized for 30 min before recording the spectrum. This procedure was repeated for the fructose and glucose samples at a concentration of 18.5%. The results are summarized in Figure 4.

From the results shown in Figure 4, it can be seen that under thermo-optic effects, the spectral shift for each type of sugar separated from the others, in contrast to the behavior when the temperature was maintained as constant and where the slopes were similar to each other, and the sugars could not be distinguished (see Figure 3). Therefore, the thermo-optic response of aqueous solutions of sugar could be used as a tool to discriminate among sugars. This stems from the fact that the thermo-optic coefficient increases with the molecular weight of the material, as it has been widely studied in glasses [47] and polymers [48]. From Figure 4b, it is evident that disaccharides (sucrose) can be clearly distinguished from monosaccharides (glucose and fructose). Surprisingly, the small difference between the thermo-optic coefficient of fructose (180.160 g/mol) and glucose (180.156 g/mol) captures the small difference in their molecular weight, which reflects the high sensitivity of the sensor presented. 

Finally, based on the results from Figure 3, a mixture of two sugars at a constant temperature would be equivalent to simply increasing the concentration of one of them. Regarding the thermo-optic effects (Figure 4), because the three sugars have slightly different responses, a mixture of two sugars will have an intermediate thermo-optic coefficient depending upon the fraction present in each sugar.

## 5. Conclusions

In summary, we have presented a practical methodology for detecting sugar concentrations in aqueous solutions. The scheme is based on an SMS fiber optics sensor, which has a simple architecture consisting of a segment of coreless MMF spliced between two standard SMFs. The MMI sensor was tested using three commercial sweeteners: sucrose, fructose, and glucose, which were controllably diluted in water. The sensitivity reported was around 0.1835 nm/wt% for sucrose, 0.1687 nm/wt% for fructose, and 0.1694 nm/wt% for glucose. The experiments indicate that sugar concentrations as small as 0.5 wt% can be detected with the OSA used. By making use of thermo-optic effects, the sugars tested could be distinguished; fundamentally, this is because of the dependence of the thermo-optic coefficient on the molecular weight of the material. This feature could be useful for quality control in industrial processes where the identification and level of concentration of sugars in the products need to be measured. In general, the results suggest that the SMS structure represents a suitable alternative both for the detection of sugar level concentrations and for the in-line quality control assessment in food processing settings. Nevertheless, it is worth noting that using controlled aqueous solutions, as in our case, serves mainly the purpose of disclosing the sensor’s performance systematically; testing the sensor on more realistic industrial liquids is yet needed to take our sensor to an application level. Furthermore, the sensor head was built without requiring functionalization or the use of special treatment on the fiber. Finally, the sensor device exhibits a simple architecture and easy fabrication, is based on commercial-grade conventional fibers, and incorporates peripheral conventional equipment. 

## Figures and Tables

**Table 1:** Standard deviations for representative samples of sugar.

Sample	Sucrose	Fructose	Glucose
6.5%	0.001 nm	0.001 nm	0.001 nm
12.5%	0.001 nm	0.001 nm	0.001 nm
18.5%	0.001 nm	0.242 nm	0.005 nm

## Data Availability

Not applicable.
